# Deficits in Cognitive Control, Timing and Reward Sensitivity Appear to be Dissociable in ADHD

**DOI:** 10.1371/journal.pone.0051416

**Published:** 2012-12-07

**Authors:** Patrick de Zeeuw, Juliette Weusten, Sarai van Dijk, Janna van Belle, Sarah Durston

**Affiliations:** Neuroimaging Lab, Developmental Disorders Unit, Department of Psychiatry, Rudolf Magnus Institute of Neuroscience, University Medical Center Utrecht, Utrecht, The Netherlands; University of Missouri-Kansas City, United States of America

## Abstract

Recent neurobiological models of ADHD suggest that deficits in different neurobiological pathways may independently lead to symptoms of this disorder. At least three independent pathways may be involved: a dorsal frontostriatal pathway involved in cognitive control, a ventral frontostriatal pathway involved in reward processing and a frontocerebellar pathway related to temporal processing. Importantly, we and others have suggested that disruptions in these three pathways should lead to separable deficits at the cognitive level. Furthermore, if these truly represent separate biological pathways to ADHD, these cognitive deficits should segregate between individuals with ADHD. The present study tests these hypotheses in a sample of children, adolescents and young adults with ADHD and controls. 149 Subjects participated in a short computerized battery assessing cognitive control, timing and reward sensitivity. We used Principal Component Analysis to find independent components underlying the variance in the data. The segregation of deficits between individuals was tested using Loglinear Analysis. We found four components, three of which were predicted by the model: Cognitive control, reward sensitivity and timing. Furthermore, 80% of subjects with ADHD that had a deficit were deficient on only one component. Loglinear Analysis statistically confirmed the independent segregation of deficits between individuals. We therefore conclude that cognitive control, timing and reward sensitivity were separable at a cognitive level and that deficits on these components segregated between individuals with ADHD. These results support a neurobiological framework of separate biological pathways to ADHD with separable cognitive deficits.

## Introduction

Heterogeneity in the clinical presentation is a key characteristic of Attention-Deficit/Hyperactivity Disorder: symptoms take many forms, ranging from subtle but pervasive attention deficits or dreaminess to impairing hyperactive, impulsive and unpredictable behavior [1]. As such, it is perhaps not surprising that efforts to clarify the underlying neurobiological substrate of the disorder have yielded considerable heterogeneity and inconsistency. To date, the endeavor to tease out a single most prominent causal factor or mechanism, be it environmental, neurocognitive, neurodevelopmental, or even genetic, has failed. As a result, investigators have shifted towards emphasizing causal heterogeneity in ADHD, where the unifying concept is that multiple neurobiological pathways may contribute to the development of overt (i.e., behavioral) and covert (i.e., neurocognitive) symptoms of the disorder [2]–[4]. An important step forward in testing the existence of multiple pathways lies in the development of falsifiable models of how dysfunction at the level of brain and cognition may lead to symptoms of ADHD 5,6.

To date, neuropsychological models of ADHD have often focused on cognitive areas with established deficits. As such, deficits in cognitive control or related concepts have been incorporated in most causal models in some form [2]–[4], [7]. Other models have emphasized timing deficits [2], or changes in sensitivity to reward or aversion to the delay of rewards [4], [8]–[10]. Theoretical accounts that stress the possibility of multiple etiological pathways to ADHD [2], [4] implicitly or explicitly suggest that affected neurocognitive functions and neurobiological systems associated with them may be separable. This has received surprisingly little attention in the neuropsychological literature on ADHD, where cognitive work addressing heterogeneity has typically investigated neuropsychological correlates of the clinical diversity of the disorder, comparing ADHD subtypes or subgroups with specific comorbidities. Recently however, investigators have begun to directly address neuropsychological heterogeneity in ADHD [11]–[13]. For example, Sonuga-Barke and colleagues recently used Principal Component Analysis (PCA) to investigate the separability of cognitive deficits in ADHD [11]. They used an extensive test battery and found separable components of inhibition, timing and delay aversion that seemed to segregate between individuals with ADHD.

Indeed, theoretical models are now emphasizing evidence from basic human and translational neuroscience supporting a neurobiological separability of brain systems affected in ADHD [5], [14]. For example, the parallel organization of distinct frontostriatal and frontocerebellar circuits forms a putative basis for biological subtypes [14]–[18]. The underlying concept is that it may be possible to translate neurobiological systems that have been associated with ADHD and that are separable at the brain level to neurobiologically defined subcategories within individuals with ADHD. Such a ‘neurobiological subtyping’ of this heterogeneous disorder would facilitate research into its neurobiological and genetic background by creating biologically more homogeneous subgroups than has proven possible with behavioral subtyping.

Researchers have begun to propose such neurobiological models of ADHD, suggesting that deficits in different neurobiological systems may lead to symptoms [2], [5], [10], [19]. We recently suggested a neurobiological framework of ADHD where deficits in separable fronto-striatal and frontocerebellar loops may lead to symptoms of ADHD [5]. Specifically, this framework predicts that deficits in cognitive control, reward processing and timing should be separable and related to neurobiological changes in dorsal frontostriatal, ventral frontostriatal and frontocerebellar networks respectively. A second important prediction of the model is that if these networks truly represent separate neurobiological systems in ADHD, then affected individuals should not be impaired on more than one system more often than would be expected by chance.

In this study, we tested some of the predictions of our model by investigating whether separate components of cognitive functions implicated in the model underlie performance on a task battery designed to test them [5]. Although we did not directly test activity of the neural circuits involved, we designed a short computerized battery from tasks that have been shown to relate to these circuits using fMRI. First, we used a modified go/no-go task that includes a timing manipulation and that has previously been validated for sensitivity to the cognitive control and timing networks. In functional MRI studies using this task, worse performance on the go/no-go manipulation was related to reduced prefrontal and frontostriatal activation in ADHD [20]–[22]. Worse performance on the timing manipulation in ADHD was related to reduced cerebellar activation and reduced corticocerebellar connectivity [20]–[23]. Second, we designed a paradigm to assess sensitivity to reward. This paradigm was a modified version of the Monetary Incentive Delay task (MID) [24], with a two-choice gambling format appropriate for children. The MID task has been shown to be sensitive to reward-related underactivation of ventral striatum in ADHD [25], [26].

Furthermore, we tested whether individuals with ADHD segregated into the subtypes predicted by the model. As our sample included subjects in a broad age range (6–27 years), we conducted exploratory analyses in a younger and an older group of subjects to investigate whether such subtyping is possible across development. One important difference from the approach by Sonuga-Barke and colleagues [11], is that we used tasks that have been shown to be relevant to the neurobiological systems targeted using functional imaging rather than tasks that are rooted in a behaviorally oriented psychological model. A second important difference is that we chose not to residualize task data for age effects, as the developmental pattern of cognitive abilities in ADHD is different from controls [27], and as such may introduce bias in the results (see also Table S1 and Figure S1). Rather, we adhered to the clinical practice of using age-based norms.

## Methods

### Participants

The study was approved by the institutional review board at the UMC Utrecht and included 63 participants with ADHD and 86 typically developing controls. After an initial screening of data integrity (see below), valid, high quality data sets were available for 57 subjects with ADHD and 83 controls. Demographic characteristics are listed in Table 1. Children and adolescents with ADHD were recruited from our outpatient clinic for disruptive disorders. Typically developing controls were recruited through schools in the wider Utrecht area. Adult subjects in both groups were tested on follow-up visits from ongoing longitudinal structural MRI research projects within the lab [28], [29].

**Table 1:** Participant characteristics.

		Controls			ADHD			Tests for group differences		
		All (n = 83)	≤12 yr (n = 48)	>12 yr (n = 35)	All (n = 57)	≤12 yr (n = 26)	>12 yr (n = 31)	All	≤12 yr	>12 yr
Gender	N Boys/Girls	60/23	32/16	28/7	47/10	19/7	28/3	.224	.610	.314
Age	M (SD)	12.7 (4.4)	9.5 (1.4)	16.9 (3.4)	12.9 (4.0)	9.6 (1.6)	15.7 (3.2)	.745	.958	.132
	Range	7.0–27.9	7.0–11.8	12.0–27.9	6.6–23.5	6.6–11.9	12.0–23.5			
Total IQ	M (SD)	111.9 (17.5)	113.7 (18.2)	109.4 (16.3)	101.4(17.4)	105.9 (19.6)	97.7 (14.5)	.001	.093	.003
	Range	76–152	78–152	76–143	72–144	72–144	73–125			
DISC-IV/MINI-Plus	ADHD Inattentive				17	6	11			
	ADHD Hyperactive/Impulsive				4	2	2			
	ADHD Combined				36	18	18			
	ODD (DISC only)				18	10	8			
CBCL/ASR^a^	Internalising raw score M (SD)^d^	4.3 (4.9)	4.5 (5.4)	3.9 (3.8)	7.7 (5.5)	8.0 (6.2)	7.5 (4.9)	<.001	.021	.005
	Externalising raw score M (SD)^d^	3.6 (3.8)	4.0 (4.2)	2.9 (3.0)	12.7 (8.6)	13.9 (8.8)	11.4 (8.5)	<.001	<.001	<.001
SES^b^	Education father (years)	13.6 (2.3)	13.4 (2.4)	14.0 (2.2)	12.5 (3.0)	12.5 (3.5)	12.6 (2.6)	.028	.275	.028
	Education mother (years)	13.3 (2.4)	13.3 (2.4)	13.4 (2.3)	12.7 (2.7)	12.7 (3.3)	12.7 (2.3)	.159	.405	.239

ADHD, Attention-Deficit/Hyperactivity Disorder; ASR, Adult Self Report; ODD, Oppositional Defiant Disorder; DISC-IV, Diagnostic Interview Schedule for Children-Fourth Edition; CBCL, Child Behavior Checklist; MINI-Plus, Mini International Neuropsychiatric Interview Plus; SES, Socio-Economic Status.

Reported are: t-tests for continuous variables, Fisher Exact test for gender (due to low cell counts and large cell count differences) and Chi^2^ for subtypes by age group (as Fisher Exact tests cannot be applied to 3×2 tables).

a. Unavailable for 11 controls and 8 subjects with ADHD.

b. Data father unavailable in 3 controls and 7 subjects with ADHD, data mother unavailable in 1 control and 7 subjects with ADHD.

### Procedure

For participants below 18 years of age (n_Control_ = 73, n_ADHD_ = 52), written informed consent was obtained from both parents after full disclosure of the study purpose and procedure. Children provided written and/or verbal assent. Parents participated in the DISC-IV, parent version [30] to confirm the clinical diagnosis of ADHD from our department (ADHD group) or to exclude psychiatric morbidity (controls). Parents filled out the Child Behavior Checklist (CBCL) [31].

Participants aged 18 years or older (n_Control_ = 10, n_ADHD_ = 5) gave written informed consent and participated in the MINI-Plus abbreviated psychiatric interview in order to confirm ADHD diagnosis or exclude psychiatric (co)morbidity [32], [33]. These subjects filled out the Adult Self Report questionnaire (the adult self-report version of the CBCL) [34]. Controls were excluded if they met DISC or MINI-Plus criteria for a psychiatric disorder or if they had first-degree relatives with a history of psychiatric problems, given the high genetic load of ADHD and its overlap with other developmental disorders. Subjects with ADHD were excluded if they met DISC-IV or MINI-Plus criteria for a comorbid disorder other than Oppositional Defiant Disorder (ODD) or Conduct Disorder (CD). First-degree family members with a history of psychiatric disorder were allowed in this group. For both groups, additional exclusion criteria were major physical or neurological disorders. According to DISC-IV scores, 17 subjects with ADHD (30%) were comorbid for ODD, and one child met criteria for both ODD and CD. The MINI-Plus does not assess ODD or CD, as they are primarily considered childhood disorders. None of our older subjects were comorbid for Antisocial Personality Disorder.

In our protocol, diagnostic interviews are repeated for subjects who participate in follow-up assessments, unless one was conducted with the last two years. As such, for subjects aged 18 years or older, the diagnostic process was based on self rather than parental report.

Subjects participated in a 1.5 hour neuropsychological assessment including an IQ assessment (WISC-III or WAIS-III short form, Dutch version) [35] and the two computerized tasks. Across studies in our lab, roughly 70–85% of Subjects with ADHD use methylphenidate medication. All participants were requested not to take any medication on the day of testing.

### Neuropsychological Battery

The first task assessed cognitive control and timing. It was a variation on a go/no-go paradigm, where go (majority) and no-go (minority) events occurred at expected regular 4 sec intervals (majority) and unexpected 2 sec intervals (minority). The paradigm has been described in detail elsewhere [20], [22]. Briefly, subjects were presented with a picture of a mouse hole on a computer screen (fixation). They were instructed to press a response button each time the door to the mouse hole opened and a piece of cheese appeared (go-trial), but to suppress their response if a cat appeared (no-go trial). Stimuli were presented for 500 ms with an inter-stimulus interval (ISI) of 3500 ms on the majority of trials. However, in 18% of trials, the stimulus was presented with an ISI of 1500 ms. As such, there were four trial types: go trials at the expected time (expected go trials; 73% of trials), no-go trials at the expected time (expected no-go trials; 9%), go trials at the unexpected time (unexpected go trials; 9%) and no-go trials at the unexpected time (unexpected no-go trials; 9%). A critical behavioral measure in the task is RT_Benefit_, which is defined as RT_Unexpected GO_ – RT_Expected GO_, and represents the response speed benefit when a trial is presented at the expected time [20], [22]. Other measures included in the PCA were accuracy on both expected and unexpected go and no-go trails (Accuracy_Expected-GO_, Accuracy_UnexpectedGO_, Accuracy_ExpectedNOGO_ and Accuracy_UnexpectedNOGO_) and Mean RT for both expected and unexpected go trials (MRT_ExpectedGO_, MRT_UnexpectedGO_). Variability in reaction times was calculated for both expected and unexpected go trials, using the Intraindividual Coefficient of Variation (ICV, ICV = SDRT/MRT) [36]. The subjects participated in four blocks of 66 trials. Task duration was 20 minutes.

The second task was adapted from the MID task to assess sensitivity to reward. As this is the first report of this task, we have included Supporting Information (Text S1, Figure S2, Figure S3, and Table S5), with a more detailed description and results comparing subjects with ADHD and controls. It is important to note that this task is still being developed and work investigating all between-group differences in depth is ongoing. Briefly, the effect of reward on reaction time was estimated in a simple two-choice gambling task, where subjects were instructed to guess which of two cartoon characters was hiding a wallet. If they guessed correctly, they earned the amount of money in the wallet. Each trial started with a 2000 ms cue indicating the amount that could be won in the upcoming trial. Reward was parametrically manipulated with three conditions, where either 0, 5 or 15 eurocents could be won. Next, both cartoon characters appeared for 750 ms. Subjects had a 1250 ms window in which to respond. They were presented with feedback stating they were “Too Late!” in a large font if they responded after this window had passed. Subjects were encouraged to guess who had the wallet even when no reward was available (0 ct trials). Four blocks of 60 trials were presented (total duration: 18 minutes). The task was rigged so that the choices made did not affect reward outcome. In the current analyses, we used data from blocks where 80% of trials were rewarded since a large part of the current sample performed an earlier version of the task in which only 80% reward frequency blocks were included. A later modification of the task employs both 20% and 80% reward frequency blocks (see Text S1, Figure S2, Table S2, Figure S3 for more details).

**Figure 1:**
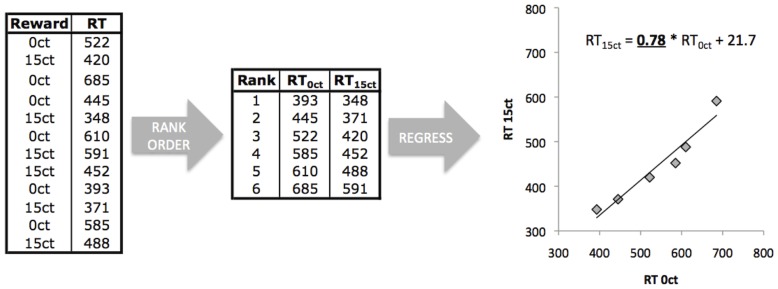
Calculation of the reaction time (RT) distribution shift measure for the reward sensitivity task. After removing accidental button presses and outliers (RT<100 ms or RT 2 SD faster or slower than the mean), RTs for each trial-type were rank ordered from fastest to slowest RT. The rank ordered RTs of the rewarded trials were then regressed on the rank ordered RTs of the non-rewarded trials for 5 ct and 15 ct conditions separately. The regression coefficients obtained (B_0vs5ct_ and B_0vs15ct_) represent the shift of the RT distribution: if B<1, the RTs in the rewarded condition were *faster*, if B>1, the RTs in the rewarded condition were *slower*.

The measure of interest from the reward task was the shift in the RT distribution from the no reward condition to the 5 ct and 15 ct conditions, calculated using linear regression of rank ordered reaction times of the reward (5, 15 ct) on the no reward (0 ct) condition. This measure was chosen as it is minimally affected by differences in intraindividual variability in RTs [37]. Figure 1 illustrates the procedure. Other measures included in the PCA were the ICV and mean RT for 0 ct trials (ICV_0 ct_ and MRT_0 ct_ respectively).

### Statistical Analyses

All statistical analyses were conducted in PASW Statistics 18. In an initial screening step, the 1.5IQR criterion was used to identify subjects that were moderate outliers on one or more measure across both tasks. Their data was used to formulate decision rules to exclude subjects who had not performed the task according to instructions. For the modified go/no-go task, this resulted in a conservative decision excluding only cases with severe disengagement from the task. Cases were excluded when response accuracy was lower than 60% on go-trials (indicating extremely low target detection) or no-go trials (indicating performance at chance level) for more than two of the four accuracy measures (Accuracy_Expected-GO_, Accuracy_UnexpectedGO_, Accuracy_ExpectedNOGO_ and Accuracy_UnexpectedNOGO_). For the reward task, outliers were mainly defined by omission trialss (i.e. no response whatsoever). Since the calculation of the RT distribution shift metrics on the reward sensitivity task required as complete as possible datasets, this resulted in an exclusion criterion where cases with an omission rate of 20% or more for any of the reward magnitude conditions (0 ct, 5 ct, 15 ct) were excluded. Using these rules, three controls and six subjects with ADHD were excluded. The exclusion rate does not differ between subjects with ADHD and controls (Fisher’s exact p = .169). Data from the resulting sample were manually inspected and cases that were at the extreme ends of the distributions of any measure were evaluated across all other measures as a final check to identify possible multivariate outliers that had been missed., No additional outliers were found in this way.

Task variables were not residualized for age prior to the Principal Component Analysis (PCA). This was based on preliminary analyses showing that age fits were not comparable across task variables and were suggestive of differential age effects between controls and subjects with ADHD (Table S1 and Figure S1).

As PCA assumes homogeneity of regression slopes between groups, we investigated this by computing correlations between all measures for both groups separately. Only the correlation between MRT_ExpectedGO_ and Accuracy_ExpectedNOGO_ was nominally different between ADHD and control subjects (p = .033). Since this was only 1 of 78 correlations computed, this did not preclude the use of PCA. There were no between-group differences in relationships between any of the task measures and age or IQ. None of the task measures correlated with IQ in either group.

All measures were entered in a PCA on the correlation matrix with the extraction criterion set at all components with an Eigenvalue >1 (a default for first-pass runs of PCA). Data from both groups were included. Initial component loadings were rotated using a Varimax rotation with Kaiser normalization in order to enhance interpretability of the component loadings [38]. A 4-component solution was extracted when the original solution based on the Eigenvalue >1 criterion yielded a fifth component that was difficult to interpret and which seemed to include only residual variance from the first component. It contained mainly MRT from both tasks and ICV from the reward task and seemed to reflect that response time was slightly modulated by instruction differences between the tasks, causing MRT on both tasks to be distributed across two components. In addition, component 5 had an Eigenvalue of only just over 1. As expected, rerunning the PCA with a 4-component solution resulted in the majority of this variance being included in the first component. As such, component scores from the 4-factor solution were saved for each individual subject and carried forward. For each of the components, we calculated the component score corresponding to the lower 10^th^ percentile of the distribution for controls. Subjects with ADHD scoring below this cut-off were defined as having a deficit on that component. Since we intended to use different norms for younger and older participants, we conducted PCAs separately for the younger and older age group, with the whole-group median age of 12 years as the split point. There were no demographic differences between the younger and older control groups, although the older ADHD group did show a trend towards lower IQ (p = .081) and had slightly fewer females than the younger ADHD group (10% versus 20%, p = .160).

We used hierarchical Loglinear Analysis to assess the statistical independence of segregation of deficits within individuals in the ADHD group [39]. This analysis is robust even with low cell counts and is more suited to testing overlap of categorical classifications with three or more categories. Chi^2^ tests can only handle sets of two categories at a time and thus cannot detect interactions (e.g., patterns of overlap in classification) between multiple categories. This analysis aims to reproduce the original data with the most parsimonious model. It begins by assuming all possible interactions between the categorical variables (the saturated model). If this is the model best to fit the data, all deficits would overlap, and all cells would be equally filled. The software then removes all interaction terms not necessary to describe the data through an iterative model building approach. At each step, a statistic (log likelihood) is calculated that indicates how well the current model fits the data. Next, the amount of log likelihood change is computed for removal of each lower order term included in the current model. The term is then removed of which the removal results in the most significant increase in log likelihood of the model (e.g. better model fit). This process continues until no more terms can be deleted (i.e., when model fit is not improved by deleting it) and as such, the most parsimonious model is found. For example, if deficits in timing and reward sensitivity were to co-occur often, a timing by reward sensitivity interaction would remain in the final model in order to describe this effect. In the case of statistical segregation of all deficits (e.g. all deficits are more likely to occur by themselves than to co-occur with other deficits), this analysis will yield a model with only the four main effects remaining.

Finally, we conducted post-hoc analyses comparing clinical measures between ADHD subjects with and without deficits our using Chi-square and independent sample t-tests as appropriate.

## Results

The results of the PCA are shown in Table 2. The measures did not cluster together, but rather segregated into four components, that we called cognitive control, timing, reward sensitivity and vigilance, based on their component loading profiles. They explained 12.6, 14.2, 10.6 and 31.6% of the variance respectively.

**Table 2:** Rotated component loadings from the PCA analysis.

	Cognitive Control			Timing			Reward Sensitivity			Vigilance		
	All	≤12 yr	>12 yr	All	≤12 yr	>12 yr	All	≤12 yr	>12 yr	All	≤12 yr	>12 yr
MRT_ExpGO_	0.016	0.331	0.205	0.300	0.167	0.402	0.028	0.017	0.263	**0.844**	**0.808**	**0.728**
MRT_UnexpectedGO_	0.041	0.308	0.104	**0.726**	**0.722**	**0.777**	0.025	0.038	0.195	**0.600**	**0.546**	**0.525**
RT_Benefit_	0.050	0.058	−0.103	**0.889**	**0.916**	**0.857**	0.004	0.038	−0.019	−0.192	−0.182	−0.077
ICV_ExpectedGO_	−0.513	−0.397	−**0.457**	0.396	0.277	0.347	0.108	0.299	−0.019	**0.459**	**0.539**	**0.620**
ICV_UnexpectedGO_	−0.296	−0.376	−0.175	**0.708**	**0.565**	**0.816**	0.007	0.044	−0.028	0.322	**0.546**	0.252
Accuracy_ExpectedGO_	0.208	0.099	0.080	−0.175	−0.016	−0.180	−0.029	−0.134	0.045	−**0.753**	−**0.796**	−**0.724**
Accuracy_UnexpectedGO_	0.174	0.138	0.021	−0.029	0.149	−0.099	−0.011	−0.041	0.022	−**0.680**	−**0.754**	−**0.733**
Accuracy_ExpectedNOGO_	**0.880**	**0.825**	**0.902**	−0.049	0.022	−0.020	−0.041	−0.070	−0.150	−0.067	0.029	−0.109
Accuracy_UnexpectedNOGO_	**0.931**	**0.877**	**0.934**	0.000	0.087	−0.006	0.027	0.054	−0.082	0.006	0.002	−0.087
B_0vs5ct_	−0.031	0.024	−0.105	0.117	0.069	0.156	**0.862**	**0.887**	**0.830**	−0.096	−0.105	−0.007
B_0vs15ct_	−0.015	0.004	−0.027	−0.083	−0.041	−0.111	**0.876**	**0.887**	**0.844**	0.057	0.110	−0.063
ICV_0ct_	−0.105	0.083	−**0.413**	0.083	−0.077	0.208	−0.034	−0.149	−0.108	−**0.415**	−0.150	−0.261
MRT_0ct_	−0.088	0.204	0.000	0.040	0.076	−0.284	−0.287	−0.302	−0.192	**0.699**	**0.647**	**0.589**
Variance explained	12.6%	16.5%	12.6%	14.2%	13.5%	16.2%	10.6%	10.8%	11.0%	31.6%	27.8%	29.5%

B, Regression Coefficient, ICV, Intra-Individual Coefficient of Variation, MRT, Mean Reaction Time, RT, Reaction Time.

Component loadings >.400 are printed in boldface.

As Table 2 shows, the PCA results were remarkably similar for the two age groups. There was a general trend for higher component loadings in the older age groups, which may well reflect developmental effects: the variance between subjects decreased with age, as many measures approached ceiling for the older group. As the PCA results were so similar between groups, we carried the results from the whole group forward to the next stage, although we computed the cut-off for deficits separately for the younger and older groups. We investigated age-related trends in the component scores by applying spline fits to the data (plots are available as Figure S1). These showed that age related changes reach a plateau level after approximately 12 years. Therefore, the age split at this point was appropriate.

None of the component scores correlated with IQ in any of the groups (Controls: all |r|<.17, p>.124, ADHD: all |r|<.16, p>.239).

**Figure 2:**
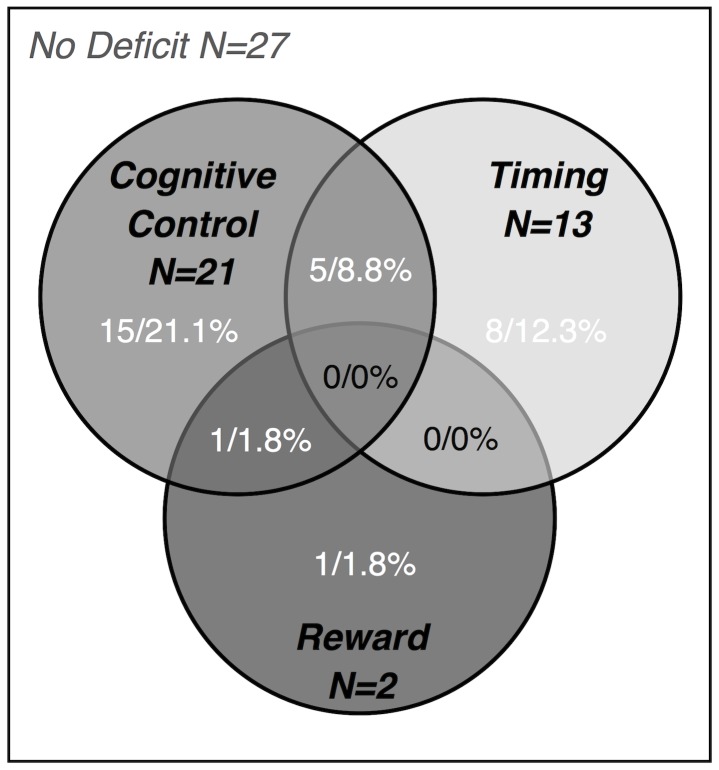
Venn diagram of deficits in the ADHD group for the predicted cognitive components.

32 Subjects with ADHD (56%) had a deficit (defined as a component score below the 10^th^ percentile worst score of the controls) on one or more component. 30 Subjects (52.6%) had deficits on at least one of the three predicted components (cognitive control, timing, and reward sensitivity). Of those, 24 (80%) had a deficit on only one component. There were no individuals with deficits on more than two components (Table 3). Figure 2 shows a Venn diagram for the components predicted by the model. To test the stability of the independence of deficits, we also computed the number of deficits using the 20^th^ and 30^th^ percentile worst score of the controls as a cutoff. By definition, more subjects with ADHD showed a deficit under the 20^th^-percentile criterion (80.7%), and more overlap between deficits was also observed. However, half of the subjects with any deficit still showed a deficit for only one component (under a 30^th^ percentile cutoff this was around 40% of subjects with ADHD with any deficit; see also Table S3 and Figure S4 for more details).

**Table 3:** Number of ADHD subjects scoring below the 10^th^ percentile of the distribution in controls.

	ADHD (Age ≤ 12 yr) (n = 26)	ADHD (Age >12 yr) (n = 31)	Whole ADHD group (n = 57)
1. Cognitive Control only	3	9	12 (21.1%)
2. Timing only	4	3	7 (12.3%)
3. Reward only	1	0	1 (1.8%)
4. Vigilance only	1	1	2 (3.5%)
5. Cognitive Control+Timing	1	4	5 (8.8%)
6. Cognitive Control+Reward	1	0	1 (1.8%)
7. Cognitive Control+Vigilance	1	2	3 (5.3%)
8. Timing+Vigilance	0	1	1 (1.8%)
9. No deficit	14	11	25 (43%)

ADHD, Attention-Deficit/Hyperactivity Disorder.

The use of the 10^th^ percentile of the control distribution as a cutoff also meant that by definition for each of the components, 10% of the control sample has a deficit. As such, under the 10^th^ percentile cutoff, we found that 27.7% of controls had a deficit on any one of the four components, a significantly smaller percentage than in the ADHD group (χ^2^ (1) = 11.45, p<.001). Of those, 82.4% has a low score on one of the predicted components only. Across a 10^th^, 20^th^, and 30^th^ percentile cutoffs, >50% of controls with any low score across all four components had such a score only for one of these components (Figure S4).

Loglinear analysis was used to test the statistical independence of the classification categories in the ADHD group (based on the 10^th^ percentile cutoff). It terminated after 11 iterations with the final model including only the main effects of cognitive control, timing, reward sensitivity and vigilance. This confirms that any overlap of these deficits within subjects can be attributed to chance.

A comparison of clinical characteristics between those subjects with ADHD who did and those who did not reach criterion for a deficit showed that subjects with a deficit had a lower total IQ (t(55) = 2.756, p<.05), and were more likely to be co-morbid for ODD (χ^2^(1) = 4.18, p<.05). There were no differences between these groups on any of the CBCL scales (all p>.179). There was no difference between clinical ADHD subtypes (combined, inattentive, hyperactive/impulsive) in mean component score on any of the four components (all p>.364), or in the proportion of subjects that showed a deficit, (χ^2^(1) = 2.49, p = .289). There was a trend towards more subjects with the inattentive subtype having a deficit on the vigilance component (23.5% of 17 subjects with inattentive subtype, compared to 5.6% of 36 subjects with combined subtype; (χ^2^(1) = 3.72, p = .054). However, we do not want to over-interpret this result given the small numbers.

## Discussion

In this study, we tested the prediction that cognitive deficits theoretically arising from different neurobiological pathways may be separable in ADHD [5]. We found four separable cognitive components, three of which we could link to the cognitive domains suggested by the model. Furthermore, loglinear analysis confirmed that deficits on these components segregated between individuals with ADHD, providing support for a multiple pathway account of ADHD [2], [4], [5]. The finding of a fourth component that we interpreted to represent vigilance was not predicted by the model, but could relate to a fourth neurobiological system involved in ADHD.

Our results converge with those from a recent study by Songa-Barke and colleagues [11]. They used a very different neuropsychological battery of nine tasks, rooted in a psychological framework rather than a battery of tasks neurobiologically validated by functional neuroimaging. They found deficits in cognitive control, timing and delay aversion that segregated between individuals with ADHD, and which bear similarity to our three predicted components (cognitive control, timing, reward sensitivity). In the current study, 80% of individuals with ADHD who had a deficit on any of these three components was deficient on only one of those components, similar to the 71% in the Sonuga-Barke dataset [11]. When all four components were considered, 69% of subjects with ADHD and a deficit had a deficit on only one component. Both our study and the study by Sonuga-Barke and colleagues therefore support the idea of separable neuropsychological subtypes in ADHD. One difference between these two studies is the proportion of subjects with ADHD who show any deficit score across the components (56% of all subjects with ADHD show one or more deficits in our data versus 71% in the report by Sonuga-Barke). This may be related to differences in the way age effects were treated: Sonuga-Barke and colleagues [11] linearly regressed out any variance associated with age from all measures prior to conducting the PCA, whereas we used separate norms for younger and older participants. For many of the variables in our study, age effects were not linear (see Table S1). As such, a regression would not have been appropriate. However, this difference could also be taken to suggest that cognitive components not measured in our battery, putatively related to other brain systems, are involved in ADHD.

In this study, we set out to test predictions of a neurobiological model in the cognitive domain. [5]. The neurobiological bases of the deficits described in the study by Sonuga-Barke and colleagues [11] are not as clear-cut: For example, both sensitivity to reward and temporal processing in the current study may be related to the construct of delay aversion in the Sonuga-Barke study. Basic neuronal temporal processing, contextual (task-related) factors and idiosyncratic factors including the perceived magnitude or emotional valence of stimuli are all known to affect the perception of how long events last [40]–[42]. As there is some evidence of a reduced response to reward in ADHD (e.g., reduced dopamine response to reward cues [8], [9]) this may be related to reports of delay aversion in this disorder, where the interval preceding reward may be perceived as longer simply due to a reduced sensitivity to reward [43]. Therefore, we explicitly aimed to separate the effects of timing and reward sensitivity.

We found only few subjects with ADHD with a deficit on the component relating to reward sensitivity. This may be related to the version of the task used, where a relatively high reward frequency schedule was applied (80%). Earlier studies have suggested that high reward frequency may reduce reward sensitivity problems in ADHD, whereas these are more obvious in designs using lower reward frequencies [9]. In a newer version of our task, we use two block types, one with relatively high and one with relatively low reward frequency. Indeed, differences between subjects with ADHD and controls are most obvious in the low reward frequency blocks (see Text S1, Figure S2, Table S2, Figure S3).

Interestingly, we found a fourth independent component in our data that was not predicted by the model and that we called vigilance. This had some face validity, as it related to both variability in RT and omission errors during go-trails. In addition, subjects with the innattentive subtype of ADHD were slightly more likely to have a deficit on this component. Subjects with a deficit on this component had a pattern of slow responding and low target detection. This is a pattern of impairments that has received relatively little attention in ADHD theory, although one model, the Cognitive Energetic Model (CEM) did underscore its relevance [3]. Empirical work has reported problems in vigilance or state regulation, particularly in studies using low event rates [44]. Our finding of an independent component corresponding to this response pattern suggests that impairments in vigilance may constitute a fourth pathway in ADHD, possibly related to attention networks [19]. Indeed, studies using Posner’s Attention Network Test, where alerting, orienting and cognitive control are separated, have also suggested that both alerting and control components may be affected in ADHD [45], [46]. These findings may also tie into recent evidence from a sibling study that suggests familial effects on cognitive impairments in ADHD separate into independent vigilance and cognitive control components [47]. In addition, some authors have identified attention networks as central to a neural systems account of ADHD (e.g. [19], [48]), based on work suggesting a possible subdivision of the dorsal attention network involved in top-down attentional control and a ventral salience attention network [48], [49].

Although there were no statistical interactions with the other three components (cognitive control, timing, reward sensitivity), impairment on the vigilance component frequently coincided with other impairments (Table 3). It is important to note that the model did not necessarily predict of impairments in vigilance would be separable from other deficits [5]. In fact, deficits in basic attention are commonly associated with subtle dysfunction in a more global attention network [19], possibly related to norepinephrine system [50]. Based on the model and the data reported here, we cannot exclude the possibility that deficits in basic arousal of varying severity may be more commonplace and not specific to any particular brain system. However, to validate such an interpretation, replication in larger samples is necessary, using targeted tasks assessing such aspects of basic attention. One hypothesis could be that the variance represented in the vigilance component would be related to a general ventral salience network rather than a dorsal attention network. As our task battery is based on fMRI-compatible event-related tasks, the event rate is lower than in typical behavioral tasks and as such may be less salient and tax vigilance more heavily. Adding a task that manipulates salience would be one way to test this hypothesis.

It is important to assess how these results map onto clinical heterogeneity in ADHD. First, in line with previous work [51], [52], we found no differences in DSM-IV ADHD subtype between those subjects who did or did not have cognitive deficits. However, co-morbid ODD was more frequent in subjects with deficits. Most earlier work has concluded that neurocognitive impairments are independent of co-morbid oppositional and aggressive symptoms, with greater ADHD symptom severity in co-morbid cases accounting for any differences [52]–[58]. This may apply here equally. The unexpected finding of lower IQ in subjects with a deficit is intriguing and converges with recent results reporting lower IQ in children with ADHD and neurocognitive deficits [57]. However, this finding could be an artifact of the slightly higher rate of deficits and a trend towards lower IQ in the older age group in our study. Furthermore, none of the neurocognitive components correlated with IQ.

We defined deficits on the cognitive components as scores falling within the bottom 10% of the control distribution. Given this definition, a number of controls were also technically characterized as having a deficit. The majority of controls with scores in the bottom 10^th^ percentile also had a deficit on one rather than multiple components (see Figure S4). Furthermore, this segregation held in both groups when other arbitrary cut-off points were used (20^th^ and 30^th^percentile; see Figure S4). Taken together, these findings add confidence that these components are truly separable.

A number of limitations are relevant to the interpretation of this study. First, the sample size is relatively modest and therefore may not be directly generalizable to all ADHD populations. Although loglinear analysis is robust at low cell counts, a larger sample would by definition have rendered the statistical analysis more robust. However, the current study converges with other recent studies, suggesting that cognitive deficits in ADHD are separable between individuals [11], [12]. This strengthens our confidence in these findings, as well as in the applicability of the approach to parsing the phenotype in ADHD. Second, we used a novel reward sensitivity task that although based on established paradigms, as yet has limited behavioral data associated with it. As such, the analyses reported here should be considered preliminary, as work in the lab is ongoing to improve both the task, as well as the characterization of how performance on this task differs for subjects with ADHD from controls. Both the preliminary nature of this characterization of task performance and the fact that no low-reward frequency blocks (see Text S1, Figure S2, Table S2, Figure S3) could be used for this study contribute to the relatively low number of subjects with a deficit on the reward component in the current dataset. Third, future work across different methodological approaches addressing neuropsychological heterogeneity in ADHD [11], [12] will critically need to address both temporal stability (e.g. test-retest reliability) and developmental (e.g., longitudinal) stability of these categories. Finally, though common in the ADHD field, we employed strict inclusion criteria and as such the generalizability of our findings to clinical reality is limited. It will be important to address how well these results hold in samples with more lenient inclusion criteria, as well as in other clinical groups with ADHD symptoms. In addition, we included subjects with varying treatment histories and as such cannot address the impact of (stimulant) treatment on these deficits.

Despite these limitations, we feel that our results come together with other studies investigating neuropsychological heterogeneity in ADHD using comparable methodology [11], [12] to support a shift towards studying ADHD from a neural systems, multiple pathway approach [19], [48], [59]. It is important to note that models suggesting causal heterogeneity usually make predictions on the level of neurobiology or cognition, i.e. on mechanical causes, or chains in the causal pathway, suggesting rather than explicating that different efficient causes (e.g., different sets of genes, environmental factors) set the different causal pathways in motion [60]. Work addressing neurobiological heterogeneity in ADHD has the potential to lead to studies of causal heterogeneity by allowing the investigation of efficient causes that may map onto separable pathways to ADHD [60].

In sum, our results support that cognitive control, timing and reward sensitivity are separable at the level of cognition and that deficits in these domains segregate between individuals with ADHD. This is in line with neurobiological models of ADHD positing that symptoms may arise from dysfunction in separate brain circuits underlying these cognitive domains. Furthermore, our data are suggestive of a fourth neurobiological pathway to ADHD involving deficits in vigilance. Such a stratification of the ADHD-phenotype into neurobiologically meaningful subtypes may facilitate future neurobiological and genetic research.

## Supporting Information

Figure S1 **Spline fits of component scores against age.** The spline fits below show the relation between age and the four components. None show linear relations with age. For Vigilance and Timing, 4^th^ order spline fits best explained the variance. For Cognitive Control and Reward, 3^rd^ order spline fits best modeled the variance.
(TIF)

Figure S2 **Task design of the reward task.** Please see Text S1 for further details.
(TIF)

Figure S3 **Between group analyses of performance on the Reward Sensitivity Task.** Please see Text S1 for further details.
(TIF)

Figure S4 **Deficit-level scoring in the ADHD and control groups across 10^th^, 20^th^ and 30^th^ percentile cutoffs.** A deficit has been defined as a score below the 10^th^ percentile worst score of controls, which is essentially an arbitrary cutoff. These figures show the results across 10^th^, 20^th^, and 30^th^ percentile cutoffs. The X-axis shows the three cutoffs, the Y-axis represents percentages. The left figure shows data for the ADHD group. The blue line shows the percentage of the total ADHD sample (n = 57) that **did not** have any component score below the cutoff. The red and green lines refer only to the subgroup that has at least one deficit (e.g. at least one component score below the X^th^ percentile). The red line shows the percentage of this group that has **only one** component score below the cutoff (e.g. only one ‘deficit’). The green line shows the percentage that has **more than one** component score below the cutoff (e.g. only one ‘deficit’). Since the cutoff method by definition categorizes a certain percentage of controls as having a deficit, the right figure shows the same data for the control group. In both groups, across these increasingly lenient cutoffs, a large percentage of the group with any deficit remains to show this for only one of the components.
(TIF)

Table S1 **To provide background on our decision not to residualize the task variables for age prior to the PCA, we report the results of linear and quadratic fits on these measures.**
(DOC)

Table S2 **Demographic characteristics of the sample reported on in Text S1.**
(DOC)

Table S3 **Deficit scores in the ADHD group at a 20^th^ percentile cutoff.**
(DOC)

Text S1 **Please see for further details.**
(DOC)
