# Supplementary figures and images for: Deficits in Cognitive Control, Timing and Reward Sensitivity Appear to be Dissociable in ADHD
Source: PLoS One. 2012 Dec 7;7(12):e51416. doi: 10.1371/journal.pone.0051416 (PMC3517570; doi:10.1371/journal.pone.0051416)

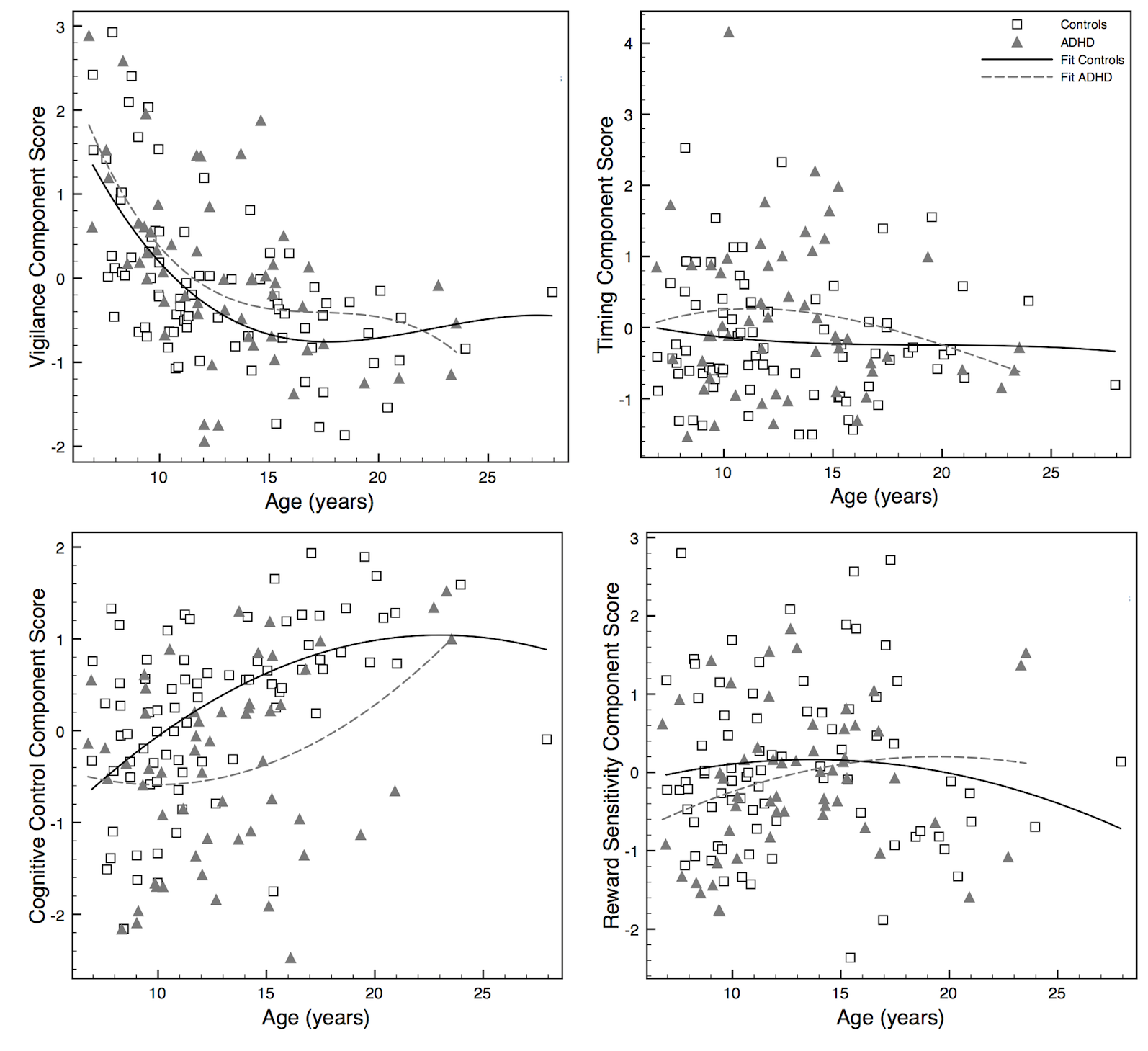

Supplement: Figure S1 — Spline fits of component scores against age. The spline fits below show the relation between age and the four components. None show linear relations with age. For Vigilance and Timing, 4th order spline fits best explained the variance. For Cognitive Control and Reward, 3rd order spline fits best modeled the variance. (TIF) [file pone.0051416.s001.tif]

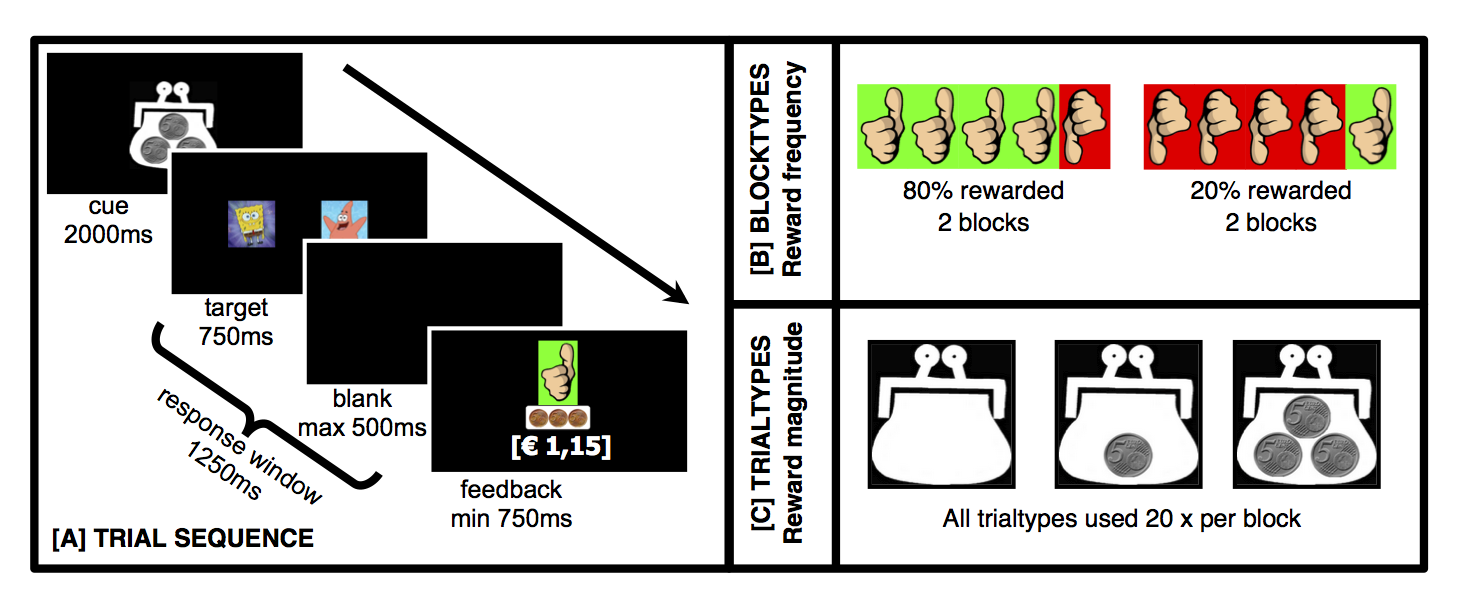

Supplement: Figure S2 — Task design of the reward task. Please see Text S1 for further details. (TIF) [file pone.0051416.s002.tif]

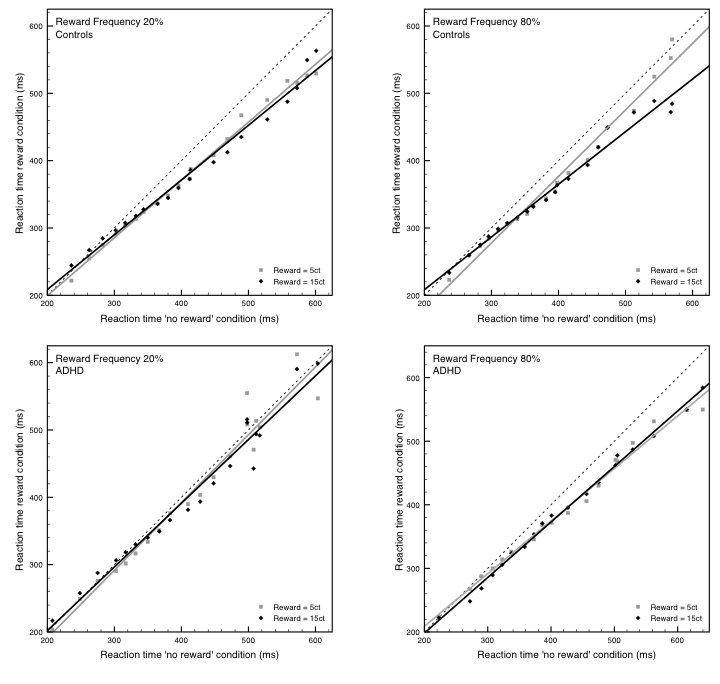

Supplement: Figure S3 — Between group analyses of performance on the Reward Sensitivity Task. Please see Text S1 for further details. (TIF) [file pone.0051416.s003.tif]

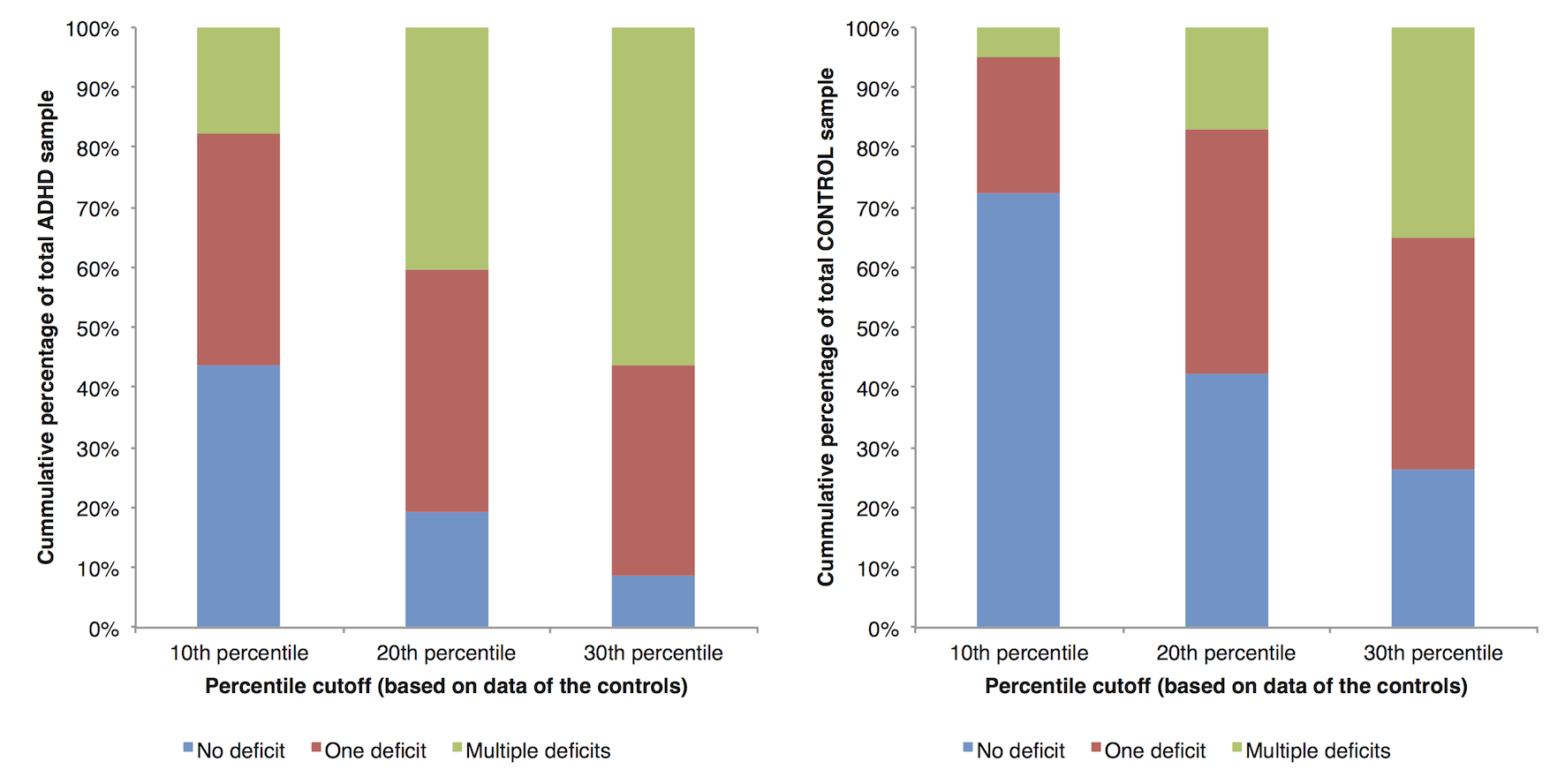

Supplement: Figure S4 — Deficit-level scoring in the ADHD and control groups across 10th, 20th and 30th percentile cutoffs. A deficit has been defined as a score below the 10th percentile worst score of controls, which is essentially an arbitrary cutoff. These figures show the results across 10th, 20th, and 30th percentile cutoffs. The X-axis shows the three cutoffs, the Y-axis represents percentages. The left figure shows data for the ADHD group. The blue line shows the percentage of the total ADHD sample (n = 57) that did not have any component score below the cutoff. The red and green lines refer only to the subgroup that has at least one deficit (e.g. at least one component score below the Xth percentile). The red line shows the percentage of this group that has only one component score below the cutoff (e.g. only one ‘deficit’). The green line shows the percentage that has more than one component score below the cutoff (e.g. only one ‘deficit’). Since the cutoff method by definition categorizes a certain percentage of controls as having a deficit, the right figure shows the same data for the control group. In both groups, across these increasingly lenient cutoffs, a large percentage of the group with any deficit remains to show this for only one of the components. (TIF) [file pone.0051416.s004.tif]
