# Supplementary material for: Deficits in Cognitive Control, Timing and Reward Sensitivity Appear to be Dissociable in ADHD
Source: PLoS One. 2012 Dec 7;7(12):e51416. doi: 10.1371/journal.pone.0051416 (PMC3517570; doi:10.1371/journal.pone.0051416)
Supplement: Table S1 — To provide background on our decision not to residualize the task variables for age prior to the PCA, we report the results of linear and quadratic fits on these measures. (DOC) [file pone.0051416.s005.doc]

**Supporting Table S1**

To provide background on our decision not to residualize the task variables for age prior to the PCA, we report the results of linear and quadratic fits on these measures. The first three columns show the results of a blocked age regression procedure across groups where we first modeled a linear age term and a quadratic term was left out of the model. When this excluded quadratic term showed significant contribution to the model it was included. For three variables, no age fit was found (e.g. the linear coefficient was not significantly different from 0). The fourth column of the table shows that trend level differences between the ADHD and control groups in these age fits were found in the data.

These analyses suggested that when the data is residualized this will strongly affect certain measures but not others. The risk is that different information is lost for certain variables at different places across the age range. In addition, residualizing will unpredictably affect the ADHD group differently than the control groups in this way, again, for some measures, but not others. Two options are then available: comparing ADHD subjects with subjects in a similar age group or residualizing the data with a different curve for each variable before conducting the PCA. Since the latter makes the results unnecessarily difficult to interpret and does not address the possibility that group effects may be partialled out in the process, we chose the former route in the main paper.

| Measure | **Whole group age fits** | | | **Group by age^a^**  (p) |
| --- | --- | --- | --- | --- |
|  | Linear (p) | Quadratic (p) | Model chosen |
| MRT_ExpGO_ | <.001 | .001 | Quadratic | .603 |
| MRT_UnexpectedGO_ | <.001 | .006 | Quadratic | .737 |
| RT_Benefit_ | - | - | No age fit | .527 |
| ICV_ExpectedGO_ | <.001 | - | Linear | .524^b^ |
| ICV_UnexpectedGO_ | <.001 | - | Linear | .093 |
| Accuracy_ExpectedGO_ | <.001 | .016 | Quadratic | .647 |
| Accuracy_UnexpectedGO_ | .012 | .053 | Quadratic | .386 |
| Accuracy_ExpectedNOGO_ | <.001 | - | Linear | .056^c^ |
| Accuracy_UnexpectedNOGO_ | <.001 | - | Linear | .218^c^ |
| B_0vs5ct_ | - | - | No age fit | .099 |
| B_0vs15ct_ | - | - | No age fit | .590 |
| ICV_0ct_ | .007 | .024 | Quadratic | .250 |
| MRT_0ct_ | <.001 | <.001 | Quadratic | .677 |

a. Reported here is the p-value for the interaction between group and age when the linear model was used or no age fit was found in the whole group curve fitting procedure (left of the table). When that procedure returned a quadratic fit as most appropriate, we the p-value of the most complex term (the group by quadratic age term) is reported.

b. When regressed on age using both a linear and quadratic term, there was a trendlevel (.05 < p < .10) interaction between group and both the linear and quadratic age term for this variable.

c. When regressed on age using both a linear and quadratic term, there was a significant (p<.05) interaction between group and both the linear and quadratic age term for this variable.

Supplemental table 2. Deficit scores at 20^th^ percentile cutoff

|  | ADHD (Age ≤ 12yr)  (n=26) | ADHD (Age > 12yr)  (n=31) | Whole ADHD group  (n=57) |
| --- | --- | --- | --- |
| 1. Cognitive Control only | 3 | 8 | 11 (19.3%) |
| 2. Timing only | 5 | 1 | 6 (10.5%) |
| 3. Reward only | 1 | 2 | 3 (5.3%) |
| 4. Vigilance only | 1 | 2 | 3 (5.3%) |
|  |  |  |  |
| Cognitive Control + Timing | 2 | 4 | 6 (10.5%) |
| Cognitive Control + Vigilance | 1 | 5 | 6 (10.5%) |
| Timing + Vigilance | 2 | 1 | 3 (5.3%) |
| Cognitive Control + Reward | 2 | 0 | 2 (3.5%) |
| Cognitive Control + Timing + Reward | 0 | 2 | 2 (3.5%) |
| Cognitive Control + Timing + Vigilance | 0 | 2 | 2 (3.5%) |
| Timing + Reward + Vigilance | 2 | 0 | 2 (3.5%) |
|  |  |  |  |
| **Any single deficit** | 10 | 13 | 23 (40.4%) |
| **Any multiple deficit** | 9 | 14 | 23 (40.4%) |
| **No deficit** | 7 | 4 | 11 (19.3%) |
