# Supplementary material for: Deficits in Cognitive Control, Timing and Reward Sensitivity Appear to be Dissociable in ADHD
Source: PLoS One. 2012 Dec 7;7(12):e51416. doi: 10.1371/journal.pone.0051416 (PMC3517570; doi:10.1371/journal.pone.0051416)
Supplement: Table S2 — Demographic characteristics of the sample reported on in Text S1. (DOC) [file pone.0051416.s006.doc]

**Supporting Table S5.** Demographic characteristics of the sample reported on in Supporting Text S3.

| **Measure** | **Control**  **n=42** | **ADHD**  **n=30** |
| --- | --- | --- |
| Age, M(SD) | 12.9(4.0) | 12.4(3.9) |
| Boys/Girls | 27/15 | 22/8 |
| TIQ, M(SD)^**^ | 114.0(19.4) | 101.1(9.5) |

ADHD, Attention-Deficit/Hyperactivity Disorder.

** p<.01
