# Supplementary material for: Deficits in Cognitive Control, Timing and Reward Sensitivity Appear to be Dissociable in ADHD
Source: PLoS One. 2012 Dec 7;7(12):e51416. doi: 10.1371/journal.pone.0051416 (PMC3517570; doi:10.1371/journal.pone.0051416)
Supplement: Table S3 — Deficit scores in the ADHD group at a 20th percentile cutoff. (DOC) [file pone.0051416.s007.doc]

**Supporting Table S6.** Deficit scores in the ADHD group at a 20^th^ percentile cutoff.

|  | **ADHD (Age ≤ 12yr)**  (n=26) | **ADHD (Age > 12yr)**  (n=31) | **Whole ADHD group**  (n=57) |
| --- | --- | --- | --- |
| 1. Cognitive Control only | 3 | 8 | 11 (19.3%) |
| 2. Timing only | 5 | 1 | 6 (10.5%) |
| 3. Reward only | 1 | 2 | 3 (5.3%) |
| 4. Vigilance only | 1 | 2 | 3 (5.3%) |
|  |  |  |  |
| Cognitive Control + Timing | 2 | 4 | 6 (10.5%) |
| Cognitive Control + Vigilance | 1 | 5 | 6 (10.5%) |
| Timing + Vigilance | 2 | 1 | 3 (5.3%) |
| Cognitive Control + Reward | 2 | 0 | 2 (3.5%) |
| Cognitive Control + Timing + Reward | 0 | 2 | 2 (3.5%) |
| Cognitive Control + Timing + Vigilance | 0 | 2 | 2 (3.5%) |
| Timing + Reward + Vigilance | 2 | 0 | 2 (3.5%) |
|  |  |  |  |
| **Any single deficit** | 10 | 13 | 23 (40.4%) |
| **Any multiple deficit** | 9 | 14 | 23 (40.4%) |
| **No deficit** | 7 | 4 | 11 (19.3%) |
