# Supplementary material for: Deficits in Cognitive Control, Timing and Reward Sensitivity Appear to be Dissociable in ADHD
Source: PLoS One. 2012 Dec 7;7(12):e51416. doi: 10.1371/journal.pone.0051416 (PMC3517570; doi:10.1371/journal.pone.0051416)
Supplement: Text S1 — Please see for further details. (DOC) [file pone.0051416.s008.doc]

**Supporting Text S3. The reward sensitivity task.**

**Please note:** This text makes reference to Supporting Figure S4 and S6 and Supporting Table S5, which are not part of this document and can be found as separate documents.

*Background*

This task was designed to estimate sensitivity to reward by measuring change in reaction times (RT) as a function of reward. RTs in choice reaction time tasks may be modulated by reward in such a way that responses are faster in the presence of reward cues . This supplement describes the task in more detail than was possible in the main paper and provides data showing between group comparisons of the effect of task manipulations.

*Task design*

The task was a simple 2-choice reaction time task that was based on the Monetary Incentive Delay (MID) task and that was adapted to be suitable for young children. It addressed only the effect of reward, rather than the effect of delay and reward. We manipulated two parameters, reward magnitude (between 0-15 Eurocents per trial) and reward frequency (20% of trials rewarded versus 80%).

The task consisted of 4 blocks of 4 minutes each, with 4 x 60 trials. Supporting Figure S4, Panel A, shows the trial sequence. Each trial started with a 2000ms cue of a wallet showing the amount of money that could be won on the upcoming trial. Next, two cartoon figures were presented. Children were instructed to guess which character was hiding the wallet, and responded by a button press on the left button for the left image and the right button for the right image. There was a 1250ms window in which to respond. The target remained on screen for 750ms, followed by a 500ms blank screen. If children responded within the 750ms window, the target remained on screen until 750 ms had passed and then went straight to the feedback screen. If children responded during the 500ms blank screen, the task jumped directly to the feedback screen. The feedback screen stated whether the guess was correct, in which case a green “thumbs up” image was shown, alongside the awarded money. If the guess was incorrect, a red “thumbs-down” image was displayed. If a child did not respond within the 1250 ms window, the feedback screen displayed “TOO LATE!” in a large font. The total accumulated reward was also displayed on the feedback screen. The feedback screen remained on screen until the full trial time of 4000ms had passed, thus for a minimum of 750ms.

The task was rigged so that it was predetermined which trials were “correct”. In other words, the choice made by the subject did not affect whether the trial was rewarded or not. This permitted experimental control of the reward frequency. There were two types of blocks, with a reward frequency of either 20% or 80% (Supporting Figure S4, Panel B). Reward magnitude was controlled, where on 1/3 of trials no reward was available (an empty wallet), on 1/3 of trials a small reward was available (5 eurocents) and on 1/3 a larger reward was available. (15 eurocents; Supporting Figure S4, Panel C). All trial types were presented 20 times per block. We used a Latin square design to ensure that all trial types were rewarded an equal number of times and that each trial type preceded every other an equal number of times during each block. This also ensured that no pattern of rewarded versus unrewarded trials was present.

Prior to the actual task, a number of instruction screens explaining the task were shown. Experimenters who administered the task explained the procedure in a standardized manner. Next, a practice block of 15 trials was administered, where 50% of trials was rewarded. The task blocks were administered either in Low-High-Low-High or High-Low-High-Low reward frequency order. The software randomly chose this order for each subject.

*Between group differences in task performance*

In order to compare the effect of the reward frequency manipulation between groups, we computed the mean RTs in each group across bins of 10 trials for 80% and 20% reward blocks separately. The dataset used here partly overlaps with the one reported in the main paper. Supporting Table S5 shows the sample characteristics.

We ran an analysis on the reward related RT distribution shift at group level, using the Regression Coefficient as outlined in the main paper (Figure 1). Here, we used the group mean RT of separate trials to calculate the measure. Supporting Figure S6 shows that controls show a distribution shift towards responding faster for both low (5ct) and high (15ct) reward magnitude in the blocks with 20% reward. This is evident from regression lines below the y=x reference (dashed line). This shift is absent in ADHD for either of the reward magnitudes under low reward frequency. For 80% reward frequency blocks, we do see a shift in children with ADHD, regardless of the reward magnitude. Controls also show a shift in the blocks with 80% reward frequency, with the greatest effect for the highest reward magnitude (15ct). These findings suggest that a higher reward frequency may “normalize” the shift in RT with reward in ADHD, in line with reward theories of ADHD.

An earlier version of the task contained only blocks with 80% reward frequency. For 32 controls (39%) and 21 subjects with ADHD (37%) in the main paper, only the earlier version of the task was available. Therefore we only included the high reward blocks in the analyses in the main paper. The pattern of responding in the 80% reward blocks was comparable between the first and second version of the task. However, as the 80% reward blocks show the smallest difference between children with ADHD and controls, this may explain any underestimation of the number of children with ADHD showing a deficit in reward sensitivity in the main paper.

References to Supporting Text S3

1. Mir P, Trender-Gerhard I, Edwards MJ, Schneider SA, Bhatia KP, et al. (2011) Motivation and movement: the effect of monetary incentive on performance speed. Exp Brain Res 209: 551-559.

2. Shadmehr R, Orban de Xivry JJ, Xu-Wilson M, Shih TY (2010) Temporal discounting of reward and the cost of time in motor control. J Neurosci 30: 10507-10516.

3. Luman M, Oosterlaan J, Sergeant JA (2005) The impact of reinforcement contingencies on AD/HD: a review and theoretical appraisal. Clinical psychology review 25: 183-213.

4. Sagvolden T, Johansen EB, Aase H, Russell VA (2005) A dynamic developmental theory of attention-deficit/hyperactivity disorder (ADHD) predominantly hyperactive/impulsive and combined subtypes. Behav Brain Sci 28: 397-419; discussion 419-368.

5. Tripp G, Wickens JR (2007) Dopamine transfer deficit: A neurobiological theory of altered reinforcement mechanisms in ADHD. J Child Psychol Psychiatry 49: 691-704.
